# Sarcopenia and mortality among a population‐based sample of community‐dwelling older adults

**DOI:** 10.1002/jcsm.12073

**Published:** 2015-10-15

**Authors:** Justin C. Brown, Michael O. Harhay, Meera N. Harhay

**Affiliations:** ^1^ Center for Clinical Epidemiology and BiostatisticsUniversity of Pennsylvania School of MedicinePhiladelphiaPAUSA; ^2^ Division of Nephrology, Department of MedicineDrexel University College of MedicinePhiladelphiaPAUSA

**Keywords:** Skeletal muscle, Body composition, Gait speed, Obesity, Population based, Cohort study

## Abstract

**Background:**

Sarcopenia is a risk‐factor for all‐cause mortality among older adults, but it is unknown if sarcopenia predisposes older adults to specific causes of death. Further, it is unknown if the prognostic role of sarcopenia differs between males and females, and obese and non‐obese individuals.

**Methods:**

A population‐based cohort study among 4425 older adults from the Third National Health and Nutrition Survey (1988–1994). Muscle mass was quantified using bioimpedance analysis, and muscle function was quantified using gait speed. Multivariable‐adjusted Cox regression analysis examined the relationship between sarcopenia and mortality outcomes.

**Results:**

The mean age of study participants was 70.1 years. The prevalence of sarcopenia was 36.5%. Sarcopenia associated with an increased risk of all‐cause mortality [hazard ratio (HR): 1.29 (95% confidence interval (95% CI): 1.13–1.47); *P* < 0.001] among males and females. Sarcopenia associated with an increased risk of cardiovascular‐specific mortality among females [HR: 1.61 (95% CI: 1.22–2.12); *P* = 0.001], but not among males [HR: 1.07 (95% CI: 0.81–1.40; *P* = .643); *P*_interaction_ = 0.079]. Sarcopenia was not associated with cancer‐specific mortality among males and females [HR: 1.07 (95% CI: 0.78–1.89); *P* = 0.672]. Sarcopenia associated with an increased risk of mortality from other causes (i.e. non‐cardiovascular and non‐cancer) among males and females [HR: 1.32 (95% CI: 1.07–1.62); *P* = 0.008]. Obesity, defined using body mass index (*P*_interaction_ = 0.817) or waist circumference (*P*_interaction_ = 0.219) did not modify the relationship between sarcopenia and all‐cause mortality.

**Conclusions:**

Sarcopenia is a prevalent syndrome that is associated with premature mortality among community‐dwelling older adults. The prognostic value of sarcopenia may vary by cause‐specific mortality and differ between males and females.

## Introduction

One of the most important changes related to ageing is the loss of muscle mass and muscle strength. Older adults lose approximately 1% of muscle mass and 3% of muscle strength each year.^1^ Sarcopenia is recognized as a geriatric syndrome characterized by critically low levels of muscle mass and muscle strength or muscle performance that predisposes individuals to adverse health outcomes.^2^, ^3^ This contemporary definition of sarcopenia evolved from a singular focus on muscle mass to a collective focus on muscle mass and muscle function,^2^, ^3^ given emerging evidence that muscle strength and muscle performance may contribute to health,^4^, ^5^, ^6^ independent of muscle mass.

Several studies have examined the relationship between low muscle mass and mortality among older adults.^7^, ^8^, ^9^, ^10^, ^11^, ^12^, ^13^ However, few studies have operationalized the definition of sarcopenia to include low muscle strength or muscle performance, in addition to low muscle mass.^14^, ^15^, ^16^ Studies conducted to date have used regression‐based anthropometric methods to estimate muscle mass,^14^, ^15^, ^16^ which may be vulnerable to error and have been discouraged from use in the assessment of sarcopenia.^2^ Many studies investigating the relationship between sarcopenia and mortality have examined all‐cause mortality. It is unknown if sarcopenia predisposes older adults to specific causes of death. It is also unknown if the relationship between sarcopenia and mortality differs between population subgroups, such as males and females,^17^ and obese and non‐obese individuals.^18^, ^19^ These subgroups may have important differences in muscle mass and muscle function, which may influence the risk of developing sarcopenia and subsequent risk of mortality.^20^

This study sought to determine if sarcopenia, defined using objective measures of muscle mass and muscle function, predicts all‐cause, cardiovascular‐specific, cancer‐specific, and other causes of premature mortality among a population‐based sample of 4425 older adults aged ≥60 years with a median of 14.4 years of follow‐up. We further aimed to determine if the relationship between sarcopenia and mortality varied between subgroups including males and females, and obese and non‐obese individuals.

## Methods

### Study population and design

The Third National Health and Nutrition Examination Survey, 1988–1994 (NHANES III) was a stratified multistage study conducted by the National Center for Health Statistics—Centers for Disease Control and Prevention, to provide health information on a nationally‐representative sample of U.S. civilians.^21^ The NHANES III sample does not include persons residing in nursing homes, members of the armed forces, institutionalized persons, or US nationals living abroad. Participants provided written informed consent prior to completing any study‐related activities. Participants in this analysis included adults of age ≥60 years with the requisite study measures necessary to define sarcopenia, as described below.

### Definition of sarcopenia

Sarcopenia was operationalized using gait speed as a measure of muscle function and bioimpedance analysis (BIA) as a measure of muscle mass.^2^, ^3^

Gait speed was assessed using a 4 m walk. Participants completed two walks, and the faster of the two trials were used in this analysis. Gait speed was calculated as the quotient of 4 m and time (in seconds) required for the walk and expressed as metres per second (m/s). Participants with a gait speed ≤0.8 m/s were considered to have a slow gait. Gait speed ≤0.8 m/s is associated with adverse health outcomes,^22^ and is recommended to identify sarcopenia.^2^

BIA was assessed using a bioresistance body composition analyser (Valhalla 1990B, Valhalla Medical, San Diego, California).^23^ Whole‐body BIA measurements were obtained between the right wrist and ankle while lying in the supine position.^23^, ^24^ All subjects were fasted for a minimum of 6 h. Muscle mass was calculated using a validated equation, where skeletal muscle mass (in kilogrammes) equals: [(height^2^ / BIA resistance × 0.401) + (gender × 3.825) + (age × −0.071)] + 5.102, where height is reported in centimetres; BIA resistance is reported in ohms; gender is equal to 1 for men and 0 for women; and age is reported in years.^25^ This equation is correlated with muscle mass quantified using magnetic resonance imaging (*r* = 0.93).^25^ The skeletal muscle index (SMI) is the absolute muscle mass (kg) indexed for height^2^ (in metres).^23^ Men with an SMI <10.76 kg/m^2^ and women with an SMI <6.75 kg/m^2^ were defined to have low muscle mass. These SMI thresholds are associated with an increased risk of disability,^23^ and are recommended to identify sarcopenia.^2^

Participants with slow gait speed (≤0.8 m/s) and low muscle mass (SMI <10.76 kg/m^2^ for men and <6.75 kg/m^2^ for women) were classified as having sarcopenia.^2^ All other participants were classified as not having sarcopenia.

### Mortality outcome

Vital status and cause of death were identified using the National Death Index (NDI) database through 31 December 2006. Participants were linked to the NDI database using probabilistic matching that included 12 identifiers such as Social Security number, sex, and date of birth.^26^ The National Center for Health Statistics found that 96.1% of deceased participants and 99.4% of living participants were correctly classified using the probabilistic matching algorithm.^27^ Cause of death was categorized using the *International Classification of Diseases 10^th^ edition* (ICD‐10).^28^ Cardiovascular‐specific mortality was categorized using ICD‐10 codes I00–I078. Cancer‐specific mortality was categorized using ICD‐10 codes C00–C97. Mortality from other causes included deaths not classified as cardiovascular‐specific or cancer‐specific. The National Center for Health Statistics removed select subject characteristics in the file to prevent re‐identification of study participants. The publically released survival data are nearly identical to the restricted‐use NHANES III mortality‐linked file.^29^

### Covariates

Demographic information including date of birth, sex, race, and education were self‐reported using a standardized questionnaire.^30^ Height (metres), body mass (kilogrammes), and waist circumference (centimetres) were measured by study technicians. Body mass index (BMI) was calculated as body mass divided by the square of height (kg/m^2^). Behavioural and clinical information including smoking status, hospitalization in the prior year, and self‐rated health were self‐reported using a standardized questionnaire.^30^ The healthy eating index was calculated from 24 h food recalls to form a score than ranges from 0 to 100 to quantify aspects of a healthy diet.^31^ Bouts of walking in the past week were self‐reported and included any bout that was estimated to be ≥1 mile in duration, and of moderate or vigorous intensity. The presence of comorbid health conditions was self‐reported by asking participants if a doctor had ever told them that they had any of the following: hypertension, diabetes, hyperlipidemia, asthma, arthritis, myocardial infarction, stroke, or congestive heart failure. Albumin, c‐reactive protein, glycated haemoglobin, insulin, glucose, and creatinine were quantified using standardized laboratory assay procedures that have been described in detail.^32^, ^33^

### Statistical analysis

The primary outcome was all‐cause mortality. Secondary outcomes included cardiovascular‐specific mortality, cancer‐specific mortality, and mortality from other causes. Continuous variables are presented as means (standard error), and categorical variables are presented as percentages (%). We used Cox proportional hazards regression models to estimate the hazard ratio [HR] and 95% confidence interval [95% CI] of sarcopenia and mortality. Models were estimated unadjusted (model 1), adjusted for sex and age (model 2), and fully adjusted for demographic, behavioural, and clinical characteristics (model 3).

The primary subgroup of interest was sex (males vs. females). The exploratory subgroup was obesity defined two ways: using BMI as a measure of general obesity [obese (BMI ≥30 kg/m^2^) vs. non‐obese (BMI < 30 kg/m^2^)]; and using waist circumference as a measure of abdominal obesity [obese (waist circumference >88 cm for women and >102 cm men) vs. non‐obese (waist circumference ≤88 cm for women and ≤102 cm men)].^34^ To determine if the relationship between sarcopenia and mortality differed between subgroups we included a statistical interaction term in the Cox proportional hazards regression models. Subgroup‐stratified analyses are presented to facilitate interpretation. As a result of the small proportion of obese (defined using either BMI or waist circumferences) persons with sarcopenia, we only examined the outcome of all‐cause mortality in the obesity subgroups. Because of the known limitations in statistical power when examining interactions,^35^ the threshold for statistical significance for interactions was *P* < 0.10 and the threshold for statistical significance for all other analyses was *P* < 0.05. All statistical analyses incorporated sample weights to account for nonresponse bias, multistage sampling probabilities, and the subpopulation of participants included in this analytic sample.^36^ Stata/SE v.13.1 statistical software was used for all analyses.

## Results

### Sarcopenia characteristics

The average age of study participants was 70.1 years old (95% CI: 69.8–70.3). Average skeletal muscle mass was 22.9 kg (95% CI: 22.6–23.2), SMI was 8.3 kg/m^2^ (95% CI: 8.2–8.3), and gait speed was 0.79 m/s (95% CI: 0.78–0.80). Age correlated with skeletal muscle mass (*r* = −0.20; *P* < 0.001), SMI (*r* = −0.17; *P* < 0.001), and gait speed (*r* = −0.33; *P* < 0.001). Gait speed correlated with skeletal muscle mass (*r* = 0.19; *P* < 0.001), and SMI (*r* = 0.13; *P* < 0.001).

**Figure 1:**
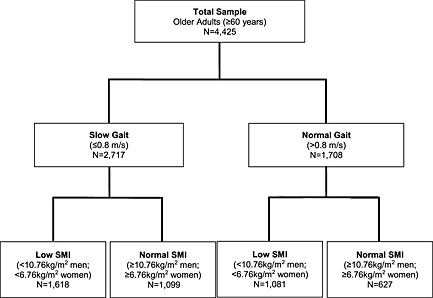
Study participant flow using sarcopenia identification algorithm. SMI: skeletal muscle index.

Among 4425 older adults, we identified 2717 individuals with slow gait speed, of whom 1618 also had a low SMI (*Figure* 1). The overall prevalence of sarcopenia was 36.5%.

### Baseline characteristics associated with sarcopenia

Characteristics of study participants are shown overall and stratified by sarcopenia status (*Table* 1).

**Table 1:** Characteristics of study participants, overall and stratified by sarcopenia status^a^

Characteristic	Overall (*N* = 4425)	Distribution of sarcopenia		*P*
	[mean (SE) or (%)]	Sarcopenia (*N* = 1618)	No sarcopenia (*N* = 2807)	
Age, yrs	70.1 (0.14)	73.1 (0.28)	68.9 (0.15)	<.001
Sex, %				
Male	43.5	53.3	39.4	<.001
Female	56.5	46.7	60.6	
Race, %				.053
White	89.5	87.3	90.4	
Black	8.3	9.7	7.7	
Other	2.2	3.0	1.9	
Education, years				
≤8	23.9	33.9	19.7	<.001
9–11	16.7	16.2	16.9	
12	31.3	29.7	33.2	
≥13	28.1	23.2	30.1	
Body mass, kg	73.7 (0.34)	67.9 (0.56)	76.1 (0.41)	<.001
Body mass index, kg/m^2^				
Continuous (mean)	27.0 (0.10)	24.7 (0.14)	27.9 (0.13)	<.001
<18.5	2.0	4.3	1.0	<.001
18.5–24.9	3.5	48.4	28.8	
25.0–29.9	39.8	37.7	40.6	
≥30	23.7	9.6	29.6	
Waist circumference, cm				
Continuous (mean)	96.9 (0.28)	93.6 (0.47)	98.3 (0.34)	<.001
≤88 (women), ≤102 (men)	42.1	54.5	37.0	<.001
>88 (women), >102 (men)	57.9	45.5	63.0	
Skeletal muscle mass, kg	22.9 (0.15)	21.6 (0.25)	23.5 (0.18)	<.001
Skeletal muscle index, kg/m^2^	8.3 (0.04)	7.8 (0.06)	8.5 (0.05)	<.001
Smoking status, %				
Never	44.2	38.7	46.5	<.001
Former	40.5	41.2	40.2	
Current	15.3	20.1	13.3	
Comorbid health conditions, %				
Hypertension	44.7	41.7	46.0	.053
Diabetes	12.2	10.0	13.1	.024
Hyperlipidemia	40.9	37.1	42.3	.047
Asthma	7.2	7.4	7.1	.843
Cancer	8.7	11.1	7.7	.006
Arthritis	44.0	45.2	43.4	.440
Heart attack	10.7	13.0	9.7	.014
Stroke	5.6	6.8	5.1	.055
Heart failure	6.0	6.9	5.6	.188
Hospitalization, %	16.1	17.3	15.6	.276
Self‐rated health, %				
Excellent	14.1	9.3	16.0	<.001
Very Good	27.7	22.7	25.5	
Good	34.3	35.0	34.1	
Fair	20.2	23.3	19.0	
Poor	6.7	9.7	5.4	
Healthy eating index	68.3 (0.29)	66.8 (0.53)	69.0 (0.34)	.001
Albumin, g/dL	4.0 (0.007)	4.1 (0.012)	4.0 (0.008)	.304
C‐reactive protein, mg/dL	0.5 (0.02)	0.6 (0.03)	0.5 (0.02)	.170
Glycated haemoglobin, %	5.8 (0.02)	5.7 (0.04)	5.8 (0.03)	.275
Insulin, pmol/L^b^	4.1 (0.01)	4.0 (0.02)	4.1 (0.02)	<.001
Glucose, mmol/L^b^	1.8 (0.005)	1.7 (0.008)	1.8 (0.006)	.310
HOMA‐insulin resistance^b^	0.9 (0.02)	0.8 (0.03)	1.0 (0.02)	<.001
Creatinine, mg/dL	1.1 (0.006)	1.2 (0.01)	1.1 (0.007)	<.001
Weekly walking, (bouts/wk)				
0	66.7	75.8	62.9	<.001
1–3	11.3	7.9	12.7	
≥3	22.0	16.3	24.4	
Gait speed, m/s	0.8 (0.005)	0.6 (0.006)	0.9 (0.005)	<.001

a Values are means (standard error) or column percentages (%).

b Variables were log‐transformed for normality.

### Sarcopenia and mortality among all participants

**Figure 2:**
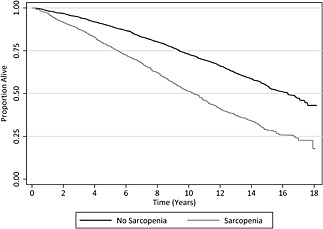
Survival of study participants, stratified by sarcopenia status.

During a median 14.4 year follow‐up period we observed 2683 deaths from all‐causes (60% of the total cohort). The median survival among subjects without sarcopenia was 16.3 years as compared with 10.3 years among subjects with sarcopenia (*P* < 0.001; *Figure* 2). In a multivariable‐adjusted regression model, sarcopenia associated with an increased risk of all‐cause mortality [HR: 1.29 (95% CI: 1.13–1.47); *P* < 0.001; (*Table* 2)].

**Table 2:** Association between sarcopenia and mortality among all participants, and stratified by sex

Mortality outcome	Hazard ratio (95% confidence interval)					
	Model 1^a^	*P*	Model 2^b^	*P*	Model 3^c^	*P*
All‐cause mortality						
All participants	2.03 (1.81–2.28)	<.001	1.40 (1.25–1.57)	<.001	1.29 (1.13–1.47)	<.001
Sex‐specific strata						
Males	1.91 (1.63–2.24)	<.001	1.42 (1.21–1.67)	<.001	1.21 (1.01–1.44)	.039
Females	2.04 (1.72–2.42)	<.001	1.37 (1.17–1.61)	<.001	1.42 (1.17–1.72)	<.001
Cardiovascular‐specific mortality						
All participants	2.19 (1.86–2.58)	<.001	1.42 (1.20–1.68)	<.001	1.34 (1.10–1.63)	.003
Sex‐specific strata						
Males	1.76 (1.39–2.23)	<.001	1.25 (0.98–1.60)	.071	1.07 (0.81–1.40)	.643
Females	2.58 (2.05–3.23)	<.001	1.60 (1.27–2.00)	<.001	1.61 (1.22–2.12)	.001
Cancer‐specific mortality						
All participants	1.46 (1.11–1.92)	.007	1.03 (0.77–1.37)	.832	1.07 (0.78–1.48)	.672
Sex‐specific strata						
Males	1.58 (1.11–2.24)	.010	1.16 (0.81–1.67)	.415	1.23 (0.81–1.89)	.336
Females	1.12 (0.69–1.82)	.633	0.86 (0.52–1.41)	.539	0.85 (0.48–1.50)	.571
Other causes of mortality						
All participants	2.10 (1.76–2.51)	<.001	1.54 (1.27–1.85)	<.001	1.32 (1.07–1.62)	.008
Sex‐specific strata						
Males	2.26 (1.77–2.90)	<.001	1.78 (1.36–2.33)	<.001	1.39 (1.05–1.84)	.022
Females	1.84 (1.41–2.41)	<.001	1.32 (1.01–1.72)	.043	1.44 (1.04–2.00)	.026

a Model 1 is unadjusted (crude).

b Model 2 is adjusted for age and sex (except in sex‐specific strata).

c Model 3 is adjusted for age, sex (except in sex‐specific strata), race, education, body mass index, waist circumference, smoking status, hypertension, diabetes, hyperlipidemia, asthma, cancer, arthritis, heart attack, stroke, and heart failure, hospitalization, self‐rated health, healthy eating index, albumin, c‐reactive protein, glycated haemoglobin, insulin (log‐transformed), glucose (log‐transformed), creatinine, and weekly bouts of walking.

We observed 1241 deaths from cardiovascular causes, 409 deaths from cancer causes, and 1033 deaths from other causes. In a multivariable‐adjusted regression model, sarcopenia associated with cardiovascular‐specific mortality [HR: 1.34 (95% CI: 1.10–1.63); *P* = 0.003], and other cause mortality [HR: 1.32 (95% CI: 1.07–1.62); *P* = 0.008], but not cancer‐specific mortality [HR: 1.07 (95% CI: 0.78–1.48); *P* = 0.672; (*Table* 2)].

### Subgroups

#### Males and females

Males and females differed in their skeletal muscle mass (29.6 vs. 17.8 kg; *P* < 0.001), SMI (9.9 vs. 7.1 kg/m^2^; *P* < 0.001), and gait speed (0.83 vs. 0.76 m/s; *P* < 0.001), respectively. The prevalence of sarcopenia was higher among males than females (35.9% vs. 24.2%; *P* < 0.001).

In multivariable‐adjusted regression models, sex did not modify the relationship between sarcopenia and all‐cause mortality (*P*_interaction_ = 0.552), cancer‐specific mortality (*P*_interaction_ = 0.238), or mortality from other causes (*P*_interaction_ = 0.637). Sex modified the relationship between sarcopenia and cardiovascular‐specific mortality (*P*_interaction_ = 0.079), such that females with sarcopenia were more likely to die from cardiovascular causes [HR: 1.61 (95% CI: 1.22–2.12); *P* = 0.001; (*Table* 2)], but sarcopenic males were not [HR: 1.07 (95% CI: 0.81–1.40); *P* = 0.643].

### Obese vs. non‐obese

**Figure 3:**
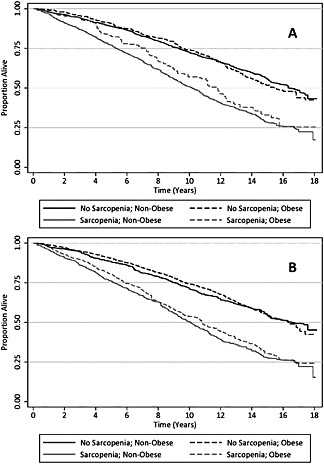
Survival of study participants, stratified by sarcopenia status and obesity, with obesity defined using: A) body mass index; and B) waist circumference.

Obese individuals (BMI‐defined) differed from non‐obese individuals in their skeletal muscle mass (25.4 vs. 22.2 kg; *P* < 0.001), SMI (9.2 vs. 8.0 kg/m^2^; *P* < 0.001), and gait speed (0.76 vs. 0.81 m/s; *P* < 0.001), respectively. BMI as a continuous variable associated with skeletal muscle mass (*r* = 0.31; *P* < 0.001), SMI (*r* = 0.43; *P* < 0.001), and gait speed (*r* = −0.08; *P* = 0.001). The prevalence of sarcopenia was lower among obese subjects compared with non‐obese subjects (34.7% vs. 11.8%; *P* < 0.001). Subjects with abdominal obesity (waist circumference‐defined) differed from those without abdominal obesity in their skeletal muscle mass (23.2 vs. 22.6 kg; *P* = 0.047), SMI (8.4 vs. 8.1 kg/m^2^; *P* = 0.001), and gait speed (0.78 vs. 0.82 m/s; *P* < 0.001), respectively. Waist circumference as a continuous variable associated with skeletal muscle mass (*r* = 0.49; *P* < 0.001), SMI (*r* = 0.52; *P* < 0.001), and gait speed (*r* = −0.06; *P* = 0.004). The prevalence of sarcopenia was lower among participants with abdominal obesity compared with those without abdominal obesity (37.8% vs. 22.9%; *P* < 0.001). Estimates of the prognostic importance of sarcopenia for all‐cause mortality were similar across obesity subgroups (*Table* 3; *Figure* 3). In the fully multivariable‐adjusted regression model, the relationship between sarcopenia and all‐cause mortality was not modified by general obesity (*P*_interaction_ = 0.817) or abdominal obesity (*P*_interaction_ = 0.219).

**Table 3:** Association between sarcopenia and all‐cause mortality, stratified by obesity status

Obesity strata	Hazard ratio (95% confidence interval)					
	Model 1^a^	*P*	Model 2^b^	*P*	Model 3^c^	*P*
General obesity (body mass index)						
Non‐obese	2.08 (1.83–2.37)	<.001	1.46 (1.29–1.65)	<.001	1.29 (1.13–1.48)	<.001
Obese	1.76 (1.31–2.37)	<.001	1.28 (0.93–1.76)	.124	1.29 (0.88–1.89)	.197
Abdominal obesity (waist circumference)						
Non‐obese	2.07 (1.74–2.47)	<.001	1.44 (1.21–1.71)	<.001	1.20 (1.01–1.45)	.044
Obese	1.96 (1.66–2.31)	<.001	1.39 (1.19–1.62)	<.001	1.45 (1.22–1.72)	<.001

a Model 1 is unadjusted (crude).

b Model 2 is adjusted for age and sex.

c Model 3 is adjusted for age, sex, race, education, smoking status, hypertension, diabetes, hyperlipidemia, asthma, cancer, arthritis, heart attack, stroke, and heart failure, hospitalization, self‐rated health, healthy eating index, albumin, c‐reactive protein, glycated haemoglobin, insulin (log‐transformed), glucose (log‐transformed), creatinine, and weekly bouts of walking.

## Discussion

The prevalence of sarcopenia in this population‐based sample of community‐dwelling older adults was 36.5%. The presence of sarcopenia associated with an increased risk of all‐cause mortality, cardiovascular‐specific mortality, and mortality from other causes. Sarcopenia was not associated with cancer‐specific mortality. Sarcopenia was associated with an increased risk of cardiovascular‐specific mortality among females, but not males. Obesity, defined using BMI or waist circumference, did not modify the prognostic importance of sarcopenia. Collectively, these data help to advance our understanding of sarcopenia among community‐dwelling older adults.

It is important to understand the strengths and weaknesses of this study to facilitate interpretation of our findings. The main strength of this study is the large sample size that, based on the sampling design, is representative of the US population of community‐dwelling older adults.^21^ Our sample included participants with a wide range of age (from 60 to 90 years). The cohort had an extensive length of follow‐up (median 14.4 years), allowing us to examine specific causes of death.

There are several limitations of this study. The primary limitation is that we did not collect handgrip strength on study participants. Handgrip strength has been endorsed in some,^2^ but not all,^3^ recommendations as a screening modality to identify persons suspected of having sarcopenia. The assessment of handgrip strength is encouraged among persons who have a normal gait speed (>0.8 m/s) to detect muscle weakness that may not be reflected in a slow gait.^2^ We relied solely on gait speed as a measure of muscle function, which is correlated with muscle strength of the lower limbs.^37^ Prior work has described that the additional cases of sarcopenia detected when handgrip strength is added to gait speed appear to be few, suggesting that gait speed alone may sufficiently discriminate those with adequate (vs. inadequate) muscle performance.^15^ A second limitation of our study is that information on comorbid health conditions was self‐reported. This likely underestimates the prevalence of comorbidities. For example, for each known case of diabetes, there exists one unknown case.^38^ The sample of obese subjects with sarcopenia was modest in the current study, and therefore our analysis that examined sarcopenia and obesity was limited to all‐cause mortality as an outcome and considered exploratory.

We found that the prevalence of sarcopenia among community‐dwelling older adults was 36.5%, a higher prevalence than previously estimated by a review conducted by the International Sarcopenia Initiative (estimated range between 1 and 29%).^39^ The heterogeneity in published estimates of sarcopenia prevalence may be influenced by multiple factors such as the age and sex distribution of the population, and the methods and cut‐points used to measure muscle mass and muscle function to define sarcopenia.^39^ In our sample age and sex were both associated with sarcopenia, such that older (vs. younger) adults and males (vs. females) were more likely to have sarcopenia. Our results are consistent with prior work defining age and sex as correlates of sarcopenia.^20^, ^39^ We used validated methods to assess muscle mass and muscle function, and recommended cut‐points to identify sarcopenia.^2^

This study identified sarcopenia as a risk factor for all‐cause mortality. We adjusted for potential confounders that included demographic, behavioural, and clinical characteristics, and sarcopenia remained a significant predictor of all‐cause mortality. This finding is consistent with prior reports documenting the deleterious effect of sarcopenia on all‐cause mortality.^14^, ^15^, ^16^ Sarcopenia was also a significant risk factor for cardiovascular‐specific mortality for females, but not males. These data substantiate earlier findings that sarcopenic females are more likely to have greater arterial stiffness than non‐sarcopenic females, an observation that was not observed among males.^40^ Sarcopenia was not associated with cancer‐specific mortality, but was associated with other‐causes of mortality (i.e. non‐cardiovascular and non‐cancer). Collectively, these data indicate that sarcopenia portends a poor prognosis among community‐dwelling older adults.

The prevalence of obesity among older adults has increased dramatically in the past three‐decades.^34^ Although sarcopenia and obesity have been hypothesized to potentiate each other causing deleterious effects on disability and mortality,^41^ our exploratory subgroup analysis of obesity did not identify any interactions of obesity on the association of sarcopenia with mortality. We defined obesity both by BMI and waist circumference, with similar findings. These data align with prior reports in males^7^ and females^10^ that obesity does not appear to modify the relationship between sarcopenia and all‐cause mortality. Nonetheless, given the high prevalence of sarcopenia and obesity among older adults, this area of research warrants additional investigation.

Sarcopenia is characterized by the age‐associated loss of skeletal muscle mass and loss of muscle function (defined by measures of muscle strength or performance).^2^, ^3^ Studies have demonstrated that muscle strength predicts mobility disability,^4^ and mortality,^5^ among older adults, independent of muscle mass. Exercise, such as slowly‐progressive weight lifting, may increase muscle strength and improve physical function among older adults.^42^ Aerobic exercise such as brisk walking is efficacious to preserve physical function among older adults.^43^ However, few studies have examined the efficacy of exercise specifically among persons with sarcopenia.^39^ The potential efficacy of exercise in this population warrants further investigation.

In summary, sarcopenia is highly prevalent among community‐dwelling older adults in the United States and is a strong prognostic factor for premature mortality among older adults. Exercise is positioned as a potentially efficacious intervention for older adults with sarcopenia. However, randomized clinical trials are necessary to demonstrate efficacy and to clarify the safety profile of exercise in this population.

## Conflict of interests

JCB, MOH, and MNH declare no conflicts of interest.
